# Automated optic disk segmentation for optic disk edema classification using factorized gradient vector flow

**DOI:** 10.1038/s41598-023-50908-5

**Published:** 2024-01-03

**Authors:** Seint Lei Naing, Pakinee Aimmanee

**Affiliations:** School of Information, Computer, and Communication Technology, Sirindhorn International Institute of Technology, Thammasat University, 131 Moo 5, Tiwanon Rd, Bangkadi, Meung, Patumthani 12000 Thailand

**Keywords:** Computer science, Biomedical engineering

## Abstract

One significant ocular symptom of neuro-ophthalmic disorders of the optic disk (OD) is *optic disk edema* (ODE). The etiologies of ODE are broad, with various symptoms and effects. Early detection of ODE can prevent potential vision loss and fatal vision problems. The texture of edematous OD significantly differs from the non-edematous OD in retinal images. As a result, techniques that usually work for non-edematous cases may not work well for edematous cases. We propose a fully automatic OD classification of edematous and non-edematous OD on fundus image collections containing a mixture of edematous and non-edematous ODs. The proposed algorithm involved localization, segmentation, and classification of edematous and non-edematous OD. The factorized gradient vector flow (FGVF) was used to segment the ODs. The OD type was classified using a linear support vector machine (SVM) based on 27 features extracted from the vessels, GLCM, color, and intensity line profile. The proposed method was tested on 295 images with 146 edematous cases and 149 non-edematous cases from three datasets. The segmentation achieves an average precision of 88.41%, recall of 89.35%, and F1-Score of 86.53%. The average classification accuracy is 99.40% and outperforms the state-of-the-art method by 3.43%.

## Introduction

**Figure 1:**
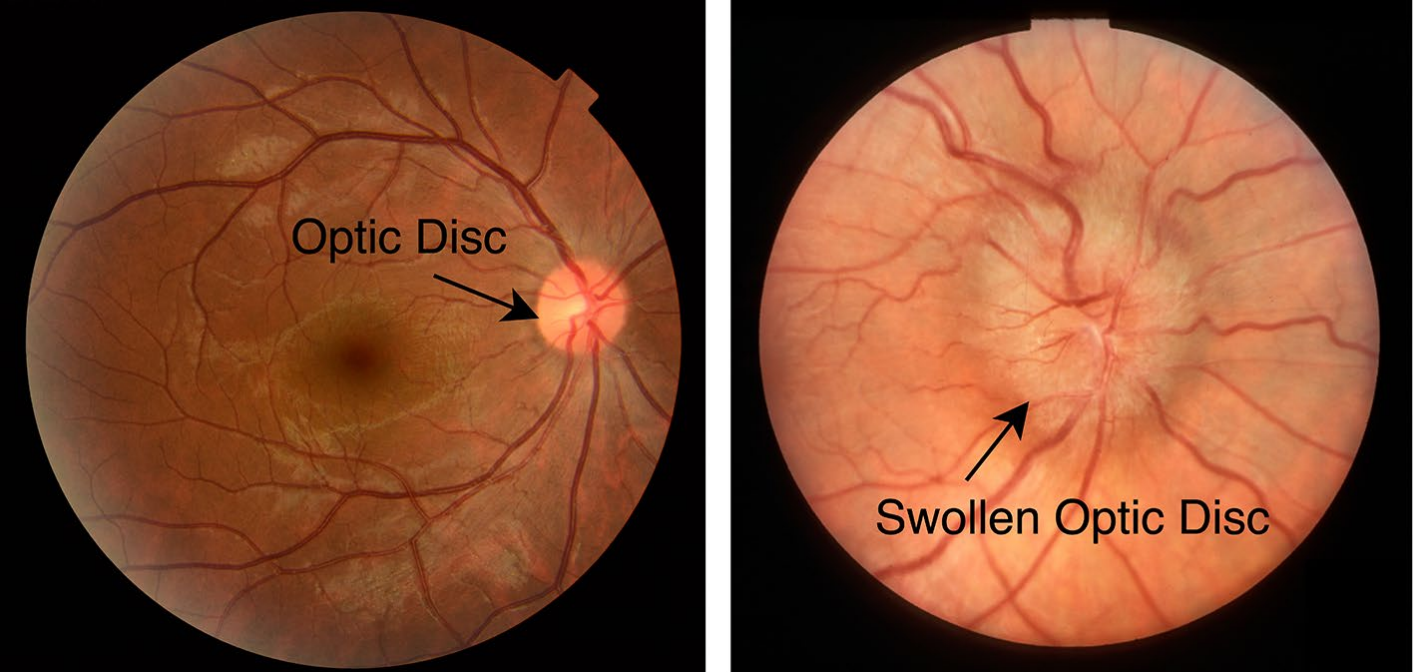
Retinal images of a non-edematous (left) and edematous (right).

Optic disk edema (ODE) is an abnormal condition describing the swelling of the optic disk. The causes of ODE are, for example, toxic optic neuropathy, infiltrative optic neuropathy, malignant hypertension, and papilledema^1^. Symptoms vary from patient to patient, depending on the causes. Common ones are eye pain, visual field loss, color vision loss, flashing lights, and even vision loss when left untreated. ODE due to idiopathic intracranial hypertension (IIH), widely known as papilledema, is the most prevalent cause of ODE. The prevalence of papilledema is as high as 3.5 out of 100,000 in females aged between 15 and 44 years old^2^. Fundus photography can be used to diagnose ODE from ophthalmic investigations. The appearances of edematous OD considerably differ from non-edematous ODs^3^. Typical characteristics of edematous OD are blur edge, disk hyperemia, elevation, peripapillary, hemorrhage, and tortuosity of retinal veins. Figure 1 shows a comparison of retinal images of non-edematous and edematous OD.

Most works for edematous OD classification are from a clinical point of view. Works related to computer-aided software or algorithms proposed for edematous OD classification purposes were limited. The following are existing works related to edematous classification, including the stage grading applicable. Deep learning and machine learning were the two main approaches used.

Milea et al.^4^ used a deep learning approach on 14,341 ocular fundus photographs, including 9156 normal retina images, 2148 papilledema images, and 3037 other retina abnormalities images for training and validation of the model and 1505 images for external testing. The system classifies images of normal, papilledema, and other abnormalities by applying U-Net for detecting OD location and DenseNet for classification. The model’s performance was evaluated by calculating the area under the curve (AUC), sensitivity, specificity, and accuracy. The overall classification performance for the detection of papilledema in the external-testing dataset is 96%, 87.5%, 96.4%, and 84.7% for AUC, accuracy, sensitivity, and specificity, respectively. Saba et al.^5^ proposed a fully automated deep-learning-based papilledema detection system using DenseNet^4^. They used DenseNet for the classification of normal and papilledema OD images. The STARE dataset with 100 images is used in the experiments. The sensitivity, specificity, accuracy, and dice coefficient of classification obtained were 98.63%, 97.83%, 99.17%, and 99.08%. Another used approach is machine learning. Fatima et al.^6^ developed a hybrid feature-based papilledema detection system. They first manually detected the OD region. The SVM classifier with thirteen extracted features from color, GLCM, statistical features, and intensity line profile was then used for classification. The method was evaluated on a small subset of the STARE dataset comprising 20 swelling cases and ten normal cases. The reported average values of the performance measures were 100% sensitivity, 95% specificity, 91.67% precision, and 96.67% accuracy, respectively. Yousaf et al.^7^ extracted six related vascular features and four GLCM textual features from 36 manually cropped non-edematous OD boundaries and ten edematous OD boundaries from the STARE dataset. The classification was performed using the supervised support vector machine (SVM) classifier with a radial basic function kernel. They reported accuracy, sensitivity, precision, and specificity measurements of individual features of 95.65%, 100%, 83.30%, and 94.40%, respectively. In both studies in the machine learning approach, the OD region used as a domain for feature extraction was manually cropped. In addition, each experiment was done on a single small dataset with an imbalance of non-edematous and edematous ODs.

As automatic OD localization and segmentation are essential steps in our work, reviews of these tasks are also provided. Many localization techniques have been proposed based on the optic disk intensity, shape, size, color, and vessel information. We have summarized the work related to OD localization in Table 1. Although several techniques with many different features have been used for OD segmentation in the past, and some even achieved accuracy as high as one hundred percent, those methods are evaluated on collections in which most images are non-edematous OD. They did not consider edematous cases. All OD localization and segmentation methods rely on typical normal ODs' intensity, shape, and size. Therefore, these methods were not suitable for edematous ODs. Thus, the accuracy may not be as good as they claimed when involved with more ODE cases. This is because of physical changes in OD appearances, such as color, brightness, and size, and associating vessel structures that tend to be incomplete and tortuous. Reviews of automatic classification, localization, and segmentation of edematous OD are provided in the next section. The summary of the techniques used for OD segmentation is shown in Table 2.

**Table 1:** Reviews on OD localization techniques.

Authors	Techniques	Datasets and sizes	Best performance %
Siddalingaswamy et al.^10^	The optimal thresholding method is used for segmenting the brightest regions, followed by connected component analysis in OD localization	148 images from the local database	Accuracy 99.3 Sensitivity 90.67 Specificity 94.06
Mahmood and Lee^11^	Using directional blur and extended maxima transform for finding OD location candidates. Images are classified into healthy and unhealthy using simple retinal image statistics to detect abnormalities. Radial blur is applied to each candidate to obtain the profiles and to distinguish the OD from other candidates	554 images from DRIVE, DIARETDB1, DIARETDB0, STARE, RIM-ONE, HAF	Accuracy 100.00
Shalchi and Rahebi^12^	The grasshopper optimization algorithm based on the light intensity of the retinal images	210 images from DIARETDB1, STARE, DRIVE	Accuracy 99.67
Devi et al.^13^	Proposed a visual attention-based OD detection system. The linear filter extracted the stimulus features from color, intensity, and orientation. The saliency model, Otsu’s thresholding combined with mathematical morphology, is mainly applied for OD detection	79 images from STARE	Accuracy 74.1
Wang et al.^14^	An integrated fully convolutional neural network (FCNN) for high-level prior knowledge and SMD model. The SLIC algorithm clusters the retinal images' superpixels, and a feature matrix is constructed with the extracted color, texture, and edge features. The generated hierarchical segmentation tree is built to measure spatial connectivity and feature similarity for a low-rank background region	182 images from DRISHTI-GS, IDRiD	Precision 94.1 Recall 96.5 F1 score 95.3
Mendonca et al.^15^	Maximum entropy calculated from the vascular network. For the low-resolution images, the intensity of the original images is considered together with the vascular maximum entropy	1361 images from DRIVE, STARE, MESSIDOR, INSPIRE-AVR	Accuracy 100.00
Soares et al.^16^	The orientation of the modified corner detector-based vessel enhancement function. The high convergence of vessels and high-intensity values defined the final OD localization	1767 images from STARE, DRIVE, DIARETDB0, DIARETDB1, MESSIDOR, ROC, E-OPHTHA-EX, HRF	Accuracy 100.00
Gui et al.^17^	An improved corner detection algorithm based on edge and gray information	1321 images from STARE, DRIVE, MESSIDOR	Accuracy 100.00
Muangnak et al.^18^	Vessel transform. The OD location is the convergence point of the directed vectors constructed from vessel segments	354 images from ROP, STARE, smartphone images database	Accuracy 98.69
Wu et al.^19^	The hybrid directional model, combines the global and local directional models. The global model uses the relationship between OD and the vascular network, whereas the local model focuses on shape, brightness, and vessel convergence	1960 images from STARE, ARIA, MESSIDOR, DIARETDB0, DIARETDB1, DRIVE, ROC, ONHSD, DRIONS	Accuracy 100.00
Zou et al.^20^	Use a verification model based on image brightness and parabolic fitting on the main vascular network	340 images STARE, DRIVE, DIARETDB0, DIARETDB1	Accuracy 100.00
Meng et al.^21^	The convolutional neural network (CNN) from red channel, green channel, and vascular information	340 images from DRIVE, DIARETDB0, DIARETDB1, STARE	Accuracy 100.00
Mahfouz and Fahmy^22^	Features projection method. An OD location is determined based on the horizontal and vertical vessel information and the intensity profile of OD features	340 images STARE, DRIVE, DIARETDB0, DIARETDB1	Accuracy 100.00
Khaing et al.^23^	The exclusion method: an extended version of the features projection method. Multiple possible OD location candidates are listed from horizontal and vertical vessel information. The final OD location is concluded using a classification based on OD features	431 images STARE, DRIVE, DIARETDB0, DIARETDB1, ROP	Accuracy 100.00
Khaing et al.^24^	Hybrid localization method. The method makes a selective model based on vascular information for applying exclusion and line models to find the OD location best	541 images from MEX, MHM, MHT, STARE, ROP, DIARETDB0, DIARETDB1	Accuracy 100.00

**Table 2:** Reviews of OD segmentation techniques.

Authors	Techniques	Dataset and size	Best performance %
Wang et al.^25^	Using the Coarse-to-fine deep learning U-Net model with Gaussian weighting used RGB color images and the vascular density map for the network weight regions	2978 images from CFI, DIARETDB0, DIARETDB1, DRIONS-DB, DRIVE, MESSIDOR, ORIGA	Jaccard 89.1, Dice 93.9, Accuracy 97.0, Sensitivity 94.4
Fang et al.^26^	Using biregional contour evolution model from the two-level set functions. The intensity, edge, and area features are considered in the method, and the Edge indicator function (EIF) is computed to differentiate OD and OC edges	1341 images from Dhristi-GS, DRIVE, REFUGE	Jaccard 93.20, Dice 96.48, Accuracy 99.77
Dashtbozorg et al.^27^	Using two sliding band filters (SBF): low-resolution SBF for initial OD center location estimation and high-resolution producing band support points for initial OD boundary	1339 images from ONHSD, MESSIDOR, INSPIRE-AVR	Overlap 89, Dice 93.73, Accuracy 99.87
Zaaboub et al.^28^	Using the saliency mask on the fundus images to localization region. Irregular shape OD boundary is refined by ellipse fitting	2050 images from RimOne, IDRID, Chase, Drive, HRF, Drishti, DRIONS, Bin Rushed, Magrabia, MESSIDOR, LocalDB	Accuracy 99.7, Dice 92.86, Jaccard 88.95, Sensitivity 97.98, Specificity 99.77
Khan et al.^29^	Using the region growing and adaptive thresholding methods. Eccentricity and size are used for the final OD selection	2054 images from DRIONS, MESSIDOR, ONHSD, DIARETDB1, DRISHTI, RIM-ONE	Sensitivity 96.49, Specificity 99.75, Accuracy 99.60,
Wilson and Mahesh^30^	Using superpixels with the k-mean algorithm	1310 images from DRIONS, MESSIDOR	Jaccard 84.23, Dice 90.84, Accuracy 99.34
Rehman et al.^31^	Using a simple linear iterative clustering algorithm technique combined with the features-based classification. The features from the segmented superpixel clusters are Intensity-based statistical features, texton-map histogram features, and fractal features, are extracted	1409 images from DRIONS, MESSIDOR, ONHSD	Sensitivity 96.9, Specificity 99.5, Dice 89.9, Accuracy 99.3
Dai et al.^32^	Using a combination of the three energies: phase-based boundary, PCA-based shape, and region energies	1409 images from MESSIDOR, ONHSD, DRIONS	Overlap 90.54
Xue et al.^33^	The hybrid level set model (HLSM) included distance-regularized, line integral and area integral, area-based, and shape-based models	138 images from DRSHTI-GS, TMUEH	Intersection over union 92.75, Four-side evaluation 464.36
Gao et al.^34^	Using saliency detection and thresholding techniques to get a rough OD boundary. The oval fitting model is used to segment higher accurate boundary	229 images from DIARETDB0, DRSHTI-GS	Overlap 66.59, Accuracy 96.30, F1-score 95.1
Abdullah et al.^35^	Using the fuzzy clustering mean method to localize the location. The active contour model is applied for OD segmentation	320 images from DRIVE, STARE, DIARETDB1, DRIONS-DB	Sensitivity 87.26, Overlap 84.56, DICE 88.40, Accuracy 99.46
Kusumandari et al.^36^	Comparing Gradient Vector Flow (GVF) snake active contour model and ellipse fitting method in OD detection	64 images from the local database	C/D ratio of area: 84.38 (GVF), 81.25 (Ellipse Fit)
Khaing et al.^37^	Using an alternated deflation-inflation gradient vector flow (ADI-GVF) model for OD and optic cup segmentation in Glaucoma prescreening application. The ADI-GVF represents a balloon model that repeatedly deflates and inflates alternately until it converges at the edge of the targeted boundary	225 images from mobile phone database, Drishti-GS, HFS	Recall 88.50, Precision 84.35, F-Measure 84.06
Gagan et al.^38^	Using basis splines-based active contour. The normalized multi-resolution-based cross-correlation (MNCC) technique is used for normalization. Gradient descent and Green’s theorem are utilized to optimize the energy function with free parameters	2993 images from Drishti-GS, MESSIDOR, RIGA, local database	Sensitivity 94.07, Specificity 99.82, Accuracy 99.71, Jaccard 85.59, Dice 93.01
Khaing et al.^8^	Using Factorized Gradient Vector Flow (FGVF) for segmentation of optic disk edema (ODE)	35 images from a public database	F1-score 84.24, Precision 91.74, Recall 79.21

## Objectives, novelty, and contributions

This study extended our previous work^8^, initially presented at the 19th International Conference on Electrical Engineering, Computer, Telecommunications, and Information Technology (ECTI-CON 2022). In that conference work, we initially introduced a factorized gradient vector flow (FGVF)^9^, a special kind of gradient vector flow for texture segmentation, to segment the edematous ODs. It is experimentally proven on a small dataset containing 35 images to yield high performance.

The extension parts from the previous work can be summarized as follows.

1. We experimented with using FGVF to segment the OD for non-edematous ODs. The previous work was only done on edematous ODs.
2. We experimented with more images from two additional public datasets containing both types of ODs. The total number of images used in the experiment is 295 with 146 edematous and 149 non-edematous cases.
3. We demonstrated that the precise OD boundary is useful for edematous OD classification.

The use of FGVF to identify the boundary of the optic disk (OD) is a groundbreaking technique in the field of research. This approach shows great promise for advancing OD detection methodologies, especially in cases where ODs are swollen. In comparison to four other state-of-the-art methods, experiments show that FGVF can provide precise OD segmentation results, regardless of whether the OD is edematous or non-edematous. This is a significant finding in the field of ophthalmic image processing, where images with mixed types of ODs are common. Moreover, the classification of OD types can be particularly useful for ODE prescreening.

## Methodology

**Figure 2:**
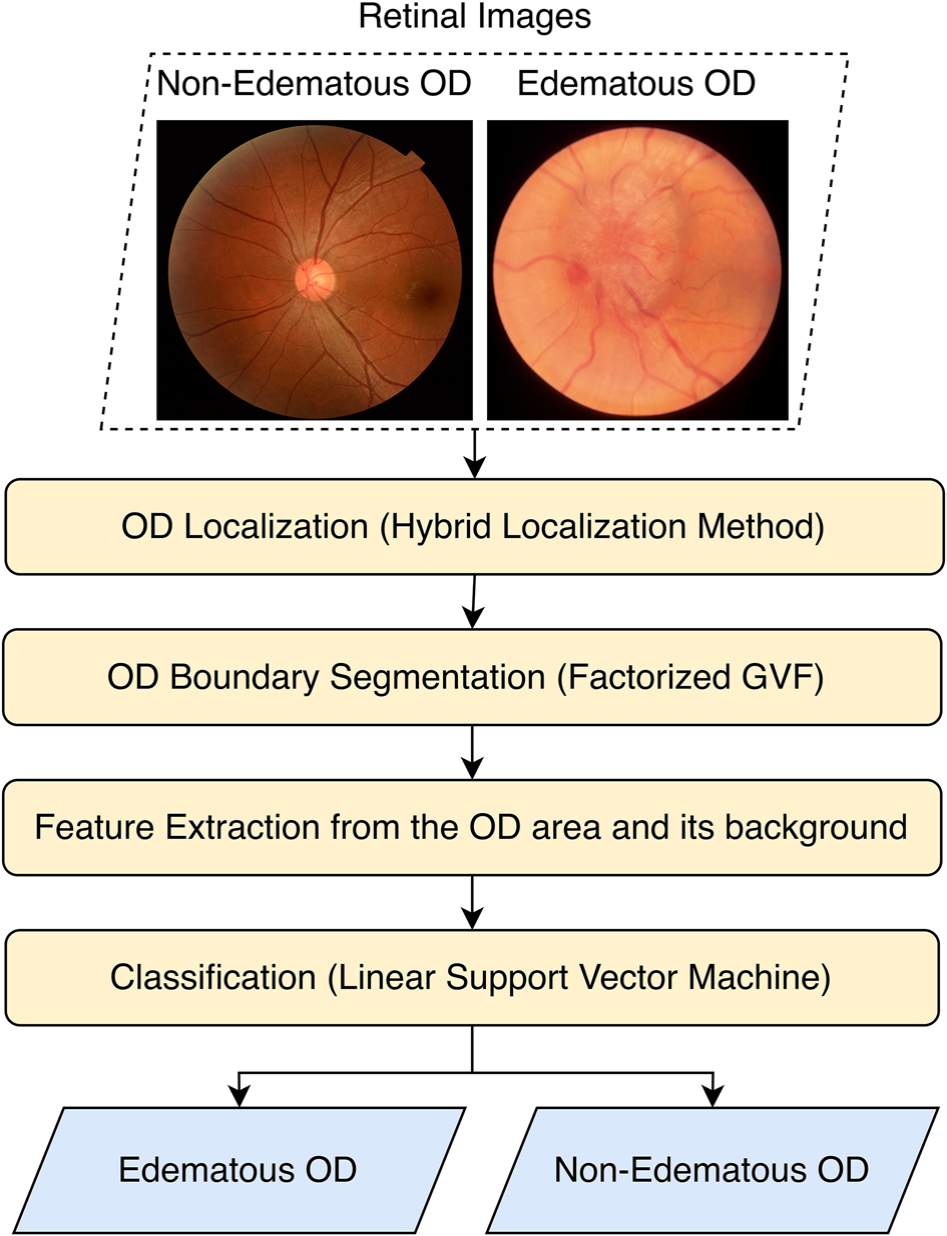
Framework of edematous and non-edematous OD classification.

A diagram depicting the procedures of our method is shown in Fig. 2. Our method comprised OD localization, OD boundary segmentation, and edematous classification. The hybrid localization method (HLM)^24^ was utilized to localize the OD. The optic disk boundary was segmented using factorized gradient vector flow (FGVF)^8,9^ with the computed location of OD used as the seed point. After OD boundary segmentation, the 27 features were extracted from a region centered at the localized OD region, and the classification of the type of OD was performed using the linear SVM classifier. The details of each step are provided in the following subsections. Settings used in SVM were described in the section Datasets, Classifiers, and Evaluation.

### OD localization

**Figure 3:**
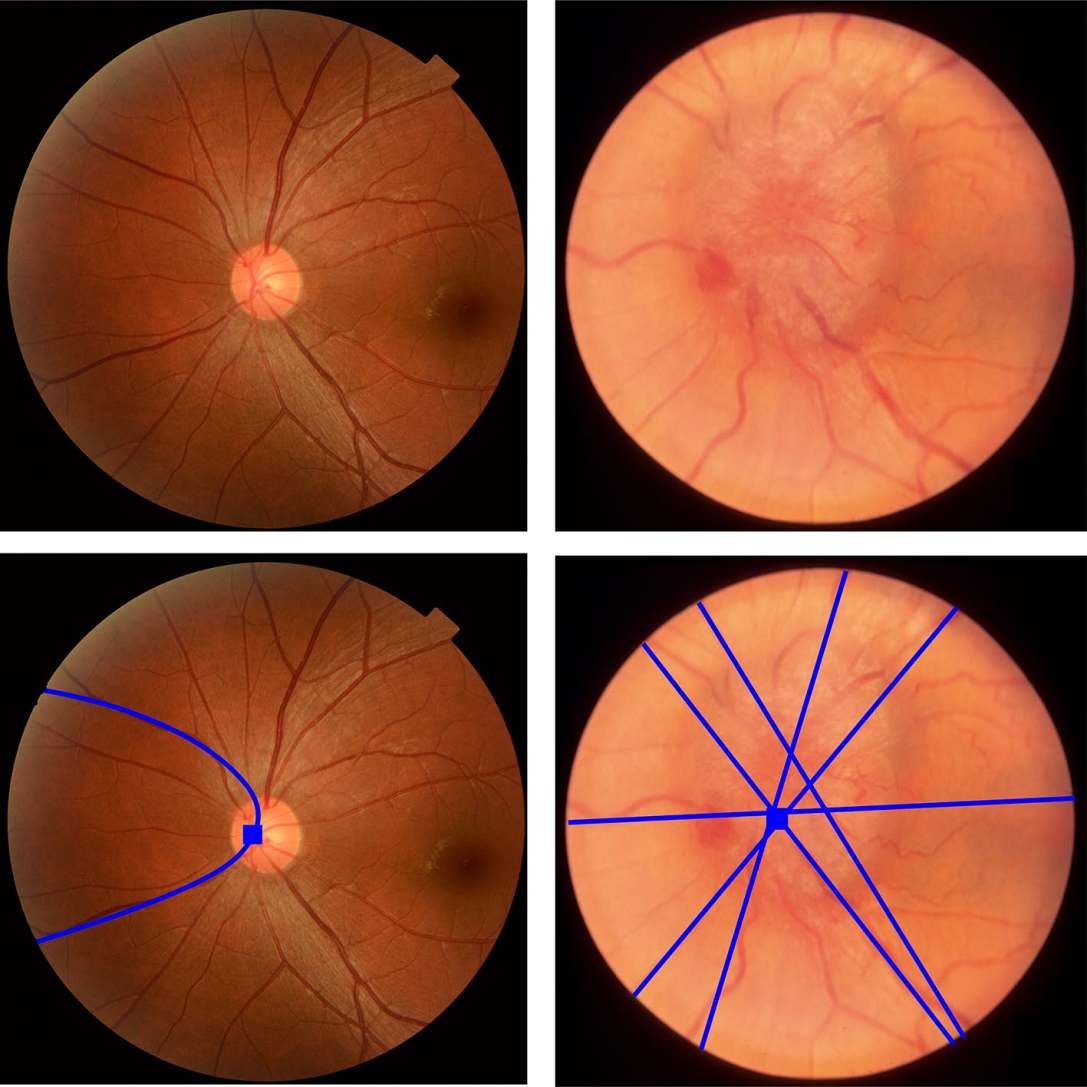
Illustration of the HLM method used for OD localization (rectangle) in non-edematous (left) and edematous ODs (right).

To locate the OD in cases where the vascular networks were incomplete, we used the hybrid localization method (HLM)^24^. This was because the vascular networks were often incomplete in edematous cases. The HLM method was effective on all types of vascular networks, regardless of their structural completeness. The HLM method first analyzed the structure of the vascular network. If the vascular network was complete, the main vessels appeared in a horizontal parabolic shape. The HLM method assumed that the vertex of the parabola was the location of the OD. If the vascular network was incomplete, it appeared as several broken lines. The OD location is determined by the convergence of the fitted straight lines, which represent these broken vessels. Figure 3 illustrates the OD localization step for edematous and non-edematous OD.

### OD boundary segmentation

**Figure 4:**
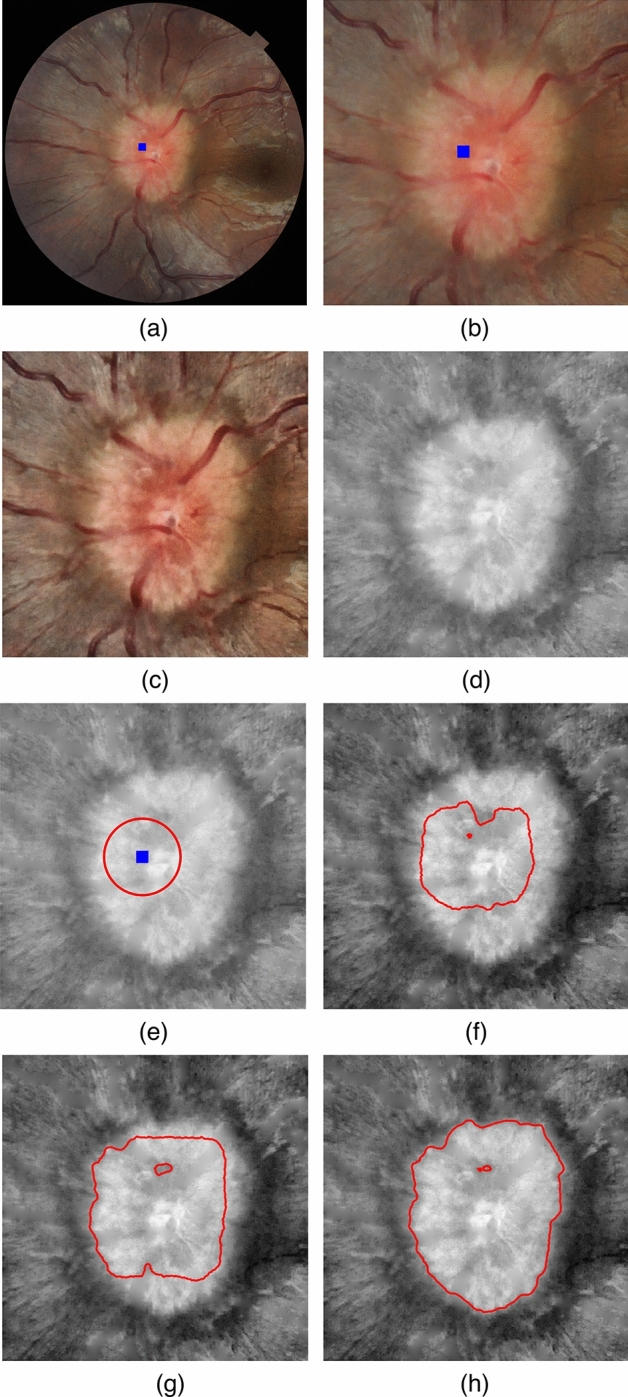
FGVF procedure illustration (**a**) Original Image, (**b**) Region of Interest (**c**) Contrast-Enhanced Image, (**d**) Vessel Removed Image, (**e**) Seed point and initial contour (**f**) after the 100th round of FGVF’s evolution process, (**g**) after the 400th round of FGVF’s evolution process, (**h**) OD boundary after FGVF’s convergence.

The OD region of interest (ROI) was first defined as a square centered at the OD location obtained from the HLM method. According to the size of OD in our datasets, we assumed that the diameter of the non-edematous OD was one-sixth of the retina’s diameter^24^. As the edematous OD’s region was commonly larger than the average size of the non-edematous OD, the ROI square’s width of both edematous and non-edematous OD was set to one-third of the retina’s diameter. Figure 4a and b depicts the original image and the ROI region.

Next, the image contrast was enhanced. The color space transform was applied to convert from an RGB to a L*a*b* channel. The Contrast-Limited Adaptive Histogram Equalization (CLAHE)^8^ was performed on the L channel on the ROI. For removing vessels from ROI, a masked image was created by multiplying the green channel of the original image with the binary ROI. Gaussian filtering was applied to the green channel of the masked image to smooth it. Then, the region filling was performed on the pixels within the mask based on Laplace’s equation, removing vessel-related regions. Figure 4c and d illustrate the results after these processes.

A factorized Gradient Vector Flow (FGVF) proposed by Gao et al.^9^ was employed in our work to segment the OD boundary. The following FGVF pseudocode illustrates the main tasks in a recursive manner. The initial contour (*C*) was defined as a circle with a radius of 1/4 of the retina's width, centered at the OD location. Subsequently, the texture feature matrix *Y* was computed from the vessel-removed image from the prior step. The FGVF algorithm takes the texture feature matrix *Y*, the initial contour *C*, the number of rounds *i* as inputs. It repeatedly evolves the contour *C* to be closer to the OD boundary until convergence.
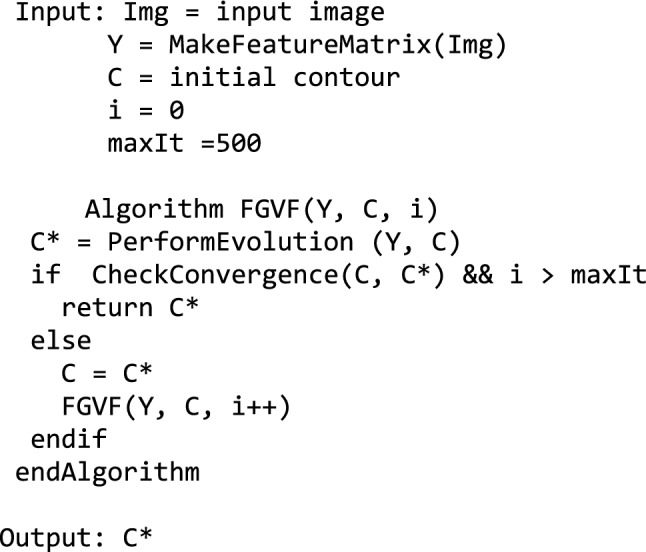


The FGVF algorithm uses the following functions.

MakeFeatureMatrix(Img) takes the image Img as an input. It returned a texture feature matrix calculated by using local spectral histograms^39^ and a factorized-based texture segmentation method proposed by Yuan et al.^40^.

PerformEvolution(Y, C) takes a texture feature matrix Y and a contour C. It evolves using the level set function and returns the new contour. The contour evolution is performed using level-set regularization proposed by Li et al.^41^.

CheckConvergence(C, C*) takes contours C and C* as inputs. If the average differences along the *x* and *y* directions between the input contours are less than a convergence threshold, the function returns true; otherwise, it returns false. In our experiment, we used 0.05 for a convergence threshold.

Figure 4e–h displays contours at the initial round, 100th round, 400th round, and upon convergence.

The contour evolution based on the factorization-based fitting energy and level-set regularization can be mathematically explained. Given $\phi$ a signed distance function of a contour curve and *R* is the presentation feature^9^.

The FGVF energy function $\left( {E_{FGVF} } \right)$ consists of two energy terms: a factorization-based fitting energy ($E_{data}$)^9^ and a level-set regularization term ($E_{regularization}$)^41^.

1 $$E_{FGVF} \left( {\phi ,R} \right) = \tau E_{data} \left( {\phi ,R} \right) + \upsilon E_{regularization} \left( \phi \right),$$

where $\tau$ and $\upsilon$ are two positive constants to control the proportion of $E_{data}$ and $E_{regularization}$. In our edematous and non-edematous OD boundary segmentation, we set the constant values $\tau$ = 50 and $\upsilon$ = 1.5. These values are tested empirically to yield the best result.

The first energy term $E_{data}$ is derived from the matrix factorization techniques. Equations (2)–(5) collectively contribute to $E_{data}$. Terms $A$ and $B$ are determined using the Heaviside function and the weight vectors $\omega_{o}$ and $\omega_{b}$. The weights are calculated from the presentation feature $R$ and the feature matrix $Y$.

2 $$E_{data} \left( {\phi , R} \right) = - \int_{\varOmega } {A + Bdx}$$

3 $$A = H_{\varepsilon } \left( \phi \right)\omega_{o} \left( {x, R} \right)$$

4 $$B = 1 - H_{\varepsilon } \left( \phi \right)\omega_{b} \left( {x, R} \right)$$

5 $$\left[ {\omega_{o} , \omega_{b} } \right] = \left( {RR^{T} } \right)^{ - 1} R^{T} Y$$

6 $$Y = R\beta + \epsilon$$

where $\varOmega$ is a 2D image domain*, x* is a point in the domain, $\varOmega_{o}$ and $\varOmega_{b}$ are defined as the object region and the background region (i.e. $\varOmega = \varOmega_{o} \cup \varOmega_{b} )$, $H_{\epsilon } \left( \phi \right)$ is a Heaviside function, $\omega_{o}$ and $\omega_{b}$ are the weights of the object and background regions, *R* is the representative features, *Y* is the feature matrix of the ROI region calculated using factorization based method for textual image segmentation proposed by Yaun et al.^40^, $\beta$ is a matrix whose columns are region weight vectors, and $\epsilon$ is the additive noise. In our work, $\epsilon$ is set to 0.5. The object and background of ROI are divided into two parts with different textural feature maps. The *Y* in Eq. (6) refers to the resultant matrix of the MakeFeatureMatrix(Img) function in the prior FGVF pseudocode.

The second energy term $E_{regularization}$ shown in Eq. (1) is expressed as:

7 $$E_{regularization} \left( \phi \right) = \int_{\varOmega } {\frac{1}{2}\left( {\left| {\nabla_{\phi } \left( x \right) - 1} \right|} \right)^{2} dx}$$

where $\nabla_{\phi }$ is the derivate of the level set function, the deformation process is repeated until the contour converges into the object boundary. Equation (7) enforces regularization and smoothness in the level-set function.

The update of the level set function at each round is described in Eq. (8).

8 $$\phi_{t + 1} = \phi_{t} + \frac{\partial \phi }{{\partial t}}dt$$

The evolving contour is a level set of $\phi$, expressed as in Eq. (9).

9 $$C = \left\{ {x :\phi \left( x \right) = 0} \right\}$$

This *C* in Eq. 9 refers to the resultant contour of the *PerformEvolution (Y, C)* in FGVF pseudocode.

## Edematous classification

A compilation of 40 different appearance-based and statistical-based features of OD was made from various literature sources. The maximum relevance minimum redundancy (mRMR) algorithm^42^ was then utilized to pick the most relevant and non-redundant features from the initial list. The mRMR algorithm prioritized features that offer informative data while minimizing any redundant information. It finally picked a set of 27 features, which we grouped into four categories, namely GLCM, vessel, color, and intensity line profiles. Below is a detailed list of the selected features.

## Gray-level co-occurrence matrix features

Gray-level co-occurrence matrix (GLCM) is a statistical technique for analyzing texture that considers the spatial relationship of pixels^43,44^. GLCM calculates the texture based on pairs of pixels with specific values and their spatial arrangement. Ten GLCM features are extracted. Let *M* be a co-occurrence matrix with *N* dimension, where $N$ is the number of gray-values, all pairs of intensities *i, j* are its coefficients and coordinates of the elements, $p$ is the normalized co-occurrence matrix, $\mu_{x} , \mu_{y}$  and σ_x_, σ_y_ are the mean and standard deviations for the matrix *p*’s rows and columns, respectively.

1. Autocorrelation (autoc) computed as the sum of the product of each element in the matrix $p$ and the product of their distance from the mean that refer to the absolute differences between the row and column indices of the element in $p$ and the mean row and column indices, respectively. It is high in edematous OD due to having similar intensity values, while non-edematous OD has lower autocorrelation because of high intensity change between optic disc and optic cup of the normal condition.
  10 $$autoc = \mathop \sum \limits_{i} \mathop \sum \limits_{j} \left( {ij} \right)p\left( {i,j} \right)$$
2. Contrast (contr) measures the difference in color shades and brightness of the region. A higher contrast value indicates a higher variation in gray level between neighboring pixels. Thus, non-edematous OD has larger contrast value than the edematous condition.
  11 $$contr = \mathop \sum \limits_{n = 0}^{{N_{g - 1} }} n^{2} \left\{ {\mathop \sum \limits_{i = 1}^{{N_{g} }} \mathop \sum \limits_{j = 1}^{{N_{g} }} p\left( {i,j} \right)\left| {\left| {i - j} \right| = n} \right.} \right\}$$
  where $n = \left| {i - j} \right|$ and $N_{g}$ is quantized gray levels.
3. Correlation (corrp) uses means and standard deviations to quantify the linear relationship between pixel intensities in the matrix $p$. The low variations in pixel intensities of the edematous case show high correlation.
  12 $$corrp = \frac{{\mathop \sum \nolimits_{i} \mathop \sum \nolimits_{j} p\left( {i,j} \right) - \mu_{x} \mu_{y} }}{{\sigma_{x} \sigma_{y} }}$$
4. Cluster prominence (cprom) measures the presence of clusters in the image, where a higher value indicates a greater prominence of clusters in the image. Thus, non-edematous OD has high value and edematous OD has low value.
  13 $$cprom = \mathop \sum \limits_{i} \mathop \sum \limits_{j} \left( {i + j - \mu_{x} - \mu_{y} } \right)^{4} p\left( {i,j} \right)$$
5. Cluster shade (cshad) measures the degree of asymmetry in the grayscale pair distribution. Non-edematous condition has high asymmetry in the distribution of the matrix $p$ and edematous case perform low asymmetry.
  14 $$cshad = \mathop \sum \limits_{i} \mathop \sum \limits_{j} \left( {i + j - \mu_{x} - \mu_{y} } \right)^{3} p\left( {i,j} \right)$$
6. Dissimilarity (dissi) measures the average absolute differences between pixel intensities in the matrix $p$. When non-edematous OD has significant changes in texture of optic cup and disc, the value is high.
  15 $$dissi = \mathop \sum \limits_{i} \mathop \sum \limits_{j} \left| {i - j} \right|.p\left( {i,j} \right)$$
7. Energy (energy) measures the uniformity of the image pixels. Texture is likely uniform in edematous OD and varying in non-edematous OD. Thus, energy value increase in abnormal disruptions of texture patterns.
  16 $$energy = \mathop \sum \limits_{i} \mathop \sum \limits_{j} p\left( {i,j} \right)^{2}$$
8. Entropy (entro) measures the disorder in the distribution of pixel pairs. The value is high when the matrix $p$’s elements are uniformly distributed. The homogenous characteristic of edematous OD condition has low entropy.
  17 $$entro = - \mathop \sum \limits_{i} \mathop \sum \limits_{j} p\left( {i,j} \right)log\left( {p\left( {i,j} \right)} \right)$$
9. Homogeneity (homop) measures image homogeneity with larger values for smaller gray tone differences in pair object. The non-edematous OD has low homogeneity compared to the edematous OD.
  18 $$homop = \mathop \sum \limits_{i} \mathop \sum \limits_{j} \frac{1}{{1 + \left( {i - j} \right)^{2} }}p\left( {i,j} \right)$$
10. Max probability (maxpr) measures the most frequently occurring intensity pair in the image. The edematous ODs tend to have a lower value of maxpr than the nonedematous.
  19 $$maxpr = Max (p\left( {i,j} \right))$$

## Vessel features

The following are the vessel features used in the experiment.

1. Vessel disk continuity Index (VDI) is the number of disjointed vessel regions in the segmented vascular network of OD images. Non-edematous OD image usually has a completely connected vascular structure, resulting in a low VDI value. In comparison, an edematous OD image usually has more broken vessels, especially in a severe case, resulting in a higher VDI value^3^.
2. Vessel disk continuity index to disk proximity (VDIP) is a VDI that calculates within the scope of the segmented OD region.
3. An area of the largest vessel region is the number of pixels in the largest vessel region. It offers details regarding the prevalence or range of the largest vessel structure. The edematous ODs tend to have a smaller number than the non-edematous ODs due to less completeness of the vascular network.
4. Mean vessel area- The ratio of the sum of vessel pixels to the total number of connected vessel clusters. Edematous ODs usually have this number lower than non-edematous ODs due to the vessel compression effect.
5. A standard deviation ($\sigma$) of the probability of intensity distribution is defined as follows.
  20 $$\sigma = \sqrt {\frac{{\sum \left( {x - \mu } \right)^{2} }}{N}} ,$$
  where *N* is the total number of pixels in the image, *x* represents each pixel intensity value, $\mu$ is the mean of image intensity distribution. The $\sigma$ values of the edematous ODs tend to be higher than the non-edematous ODs.
6. Kurtosis distribution ($\kappa$) is a measure of the tailedness of an intensity distribution defined as follows.
  21 $$\kappa = \frac{{\frac{1}{N}\sum \left( {x - \mu } \right)^{4} }}{{\sigma^{4} }}$$
  where *N* is the total number of pixels in the image, *x* represents each pixel intensity value, $\mu$ is the mean of image intensity distribution, and $\sigma$ is the standard deviation. It indicates how often the outliers occur.

The $\kappa$ values of the edematous ODs tend to be lower than the non-edematous ODs.

## Color features

1. Sharpness (*S*): the ratio of the sum of all gradient norms and the number of image pixels.
2. The hue value (*H*) in the HSV space
3. The saturation value (*S*) in the HSV space
4. The brightness (*V*) in the HSV space
5. Mean values of the intensity
6. The Red/Green value (a*) in the L*a*b* color spaces
7. The Blue/Yellow value (b*) in the L*a*b* color spaces

Generally, these color features of non-edematous ODs are higher than edematous ODs.

## Image intensity line profile features

**Figure 5:**
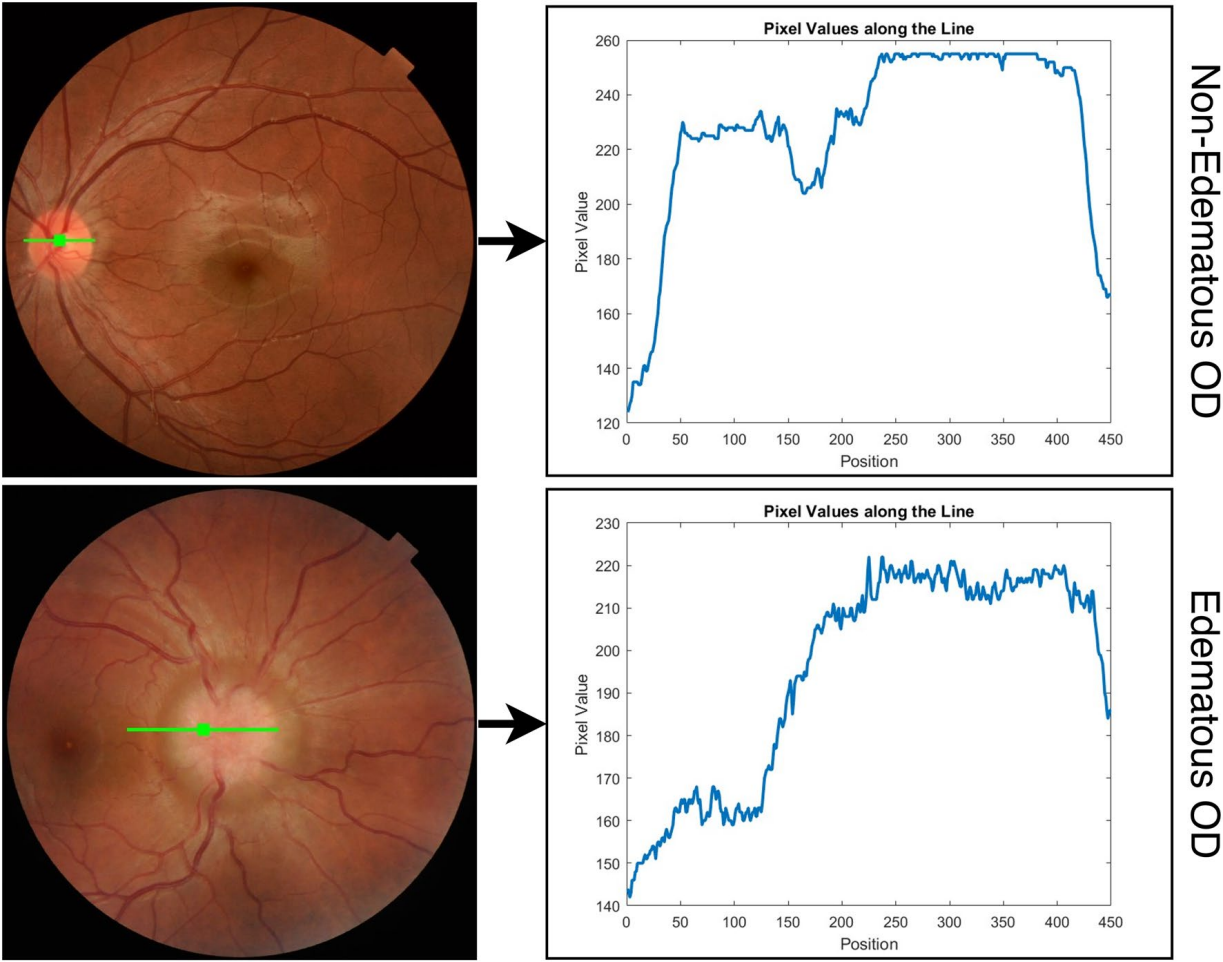
Example of image intensity profile from a line on edematous and non-edematous OD image.

A horizontal line centered at the optic disk (OD) location with a length one-half of the diameter of the retina is considered. Figure 5 depicts the intensity profile.

The following features are extracted from a line profile.

1. The average intensity
2. The minimum intensity
3. The maximum intensity
4. The standard deviation of intensity

Generally, the averages, the maximums, and the standard deviations of the intensity of the non-edematous ODs are larger than the edematous ODs. In contrast, the minimum intensity values of non-edematous ODs are lower than those of edematous ODs.

## Datasets, classifier, and evaluation

The programs were implemented using MATLAB R2022a and ran on DELL IN5406 (Intel Core i7-1165G7 Processor). The experiments were tested on three datasets. The first dataset downloaded images from the Internet^45,46^ includes 35 edematous and 38 non-edematous ODs images with the dimensions between 600 × 600 and 2300 × 1900. The selected fundus images with optic disk edema from RFMiD public dataset^47^ contained 91 edematous and 91 non-edematous ODs images with dimensions between 2144 × 1424 and 4288 × 2848. From the RFMiD2.0 public dataset^48^, 20 edematous and 20 non-edematous OD images with the dimensions 512 × 512 and 2048 × 1536 were selected. A total of 295 OD images with 146 edematous and 149 non-edematous cases were used in the experiments.

For the ODE classification, we selected a Linear Support Vector Machine (SVM) since it is effective with datasets with many features. To minimize over-fitting, we used fivefold cross-validation approach with 80% training and 20% testing. However, when dealing with a new image with different characteristics from the current dataset, over-fitting may still occur. Additionally, it is important to note that there are limited publicly available retinal images with edematous ODs. Thus, it is currently not possible to solve the issue of over-fitting by simply increasing the size of the dataset.

For OD localization, the performance was measured using *a* location accuracy* (Acc*_*loc*_) defined in Eq. (22).

22 $$Acc_{loc} = \frac{C}{N},$$

where *C* is the number of images the method correctly localizes the OD, and *N* is the number of images. Remark that the case is successful when the method’s calculated OD location is within the ground truth contour.

The performance of the OD segmentation method was evaluated using precision, recall, and F1 measures. The evaluation formulas are shown in Eqs. (23)–(25).

23 $$Precision = \frac{TP}{{TP + FP}},$$

24 $$Recall = \frac{TP}{{TP + FN}},$$

25 $$F1 \, measure = \frac{2 \times Precision \times Recall}{{Precision + Recall}},$$

where *TP, FP, TN,* and *FN* are the number of pixels that are true positive, false positive, true negative, and false negative, respectively.

For edematous classification, we compared the performances of each feature and all together features using a support vector machine (SVM) linear classifier. The classification accuracy* (Acc*_*classify*_) is defined in Eq. (26).

26 $$Acc_{classify} = \frac{{C_{Ede} + C_{Non} }}{N},$$

where $C_{Ede}$ and $C_{Non}$ are the numbers of images correctly classified as edematous and non-edematous and N is the number of images.

## Numerical results and discussion

This section presents comparative and quantitative studies of localization, segmentation, and classification of edematous OD compared to the existing methods.

### OD localization

**Figure 6:**
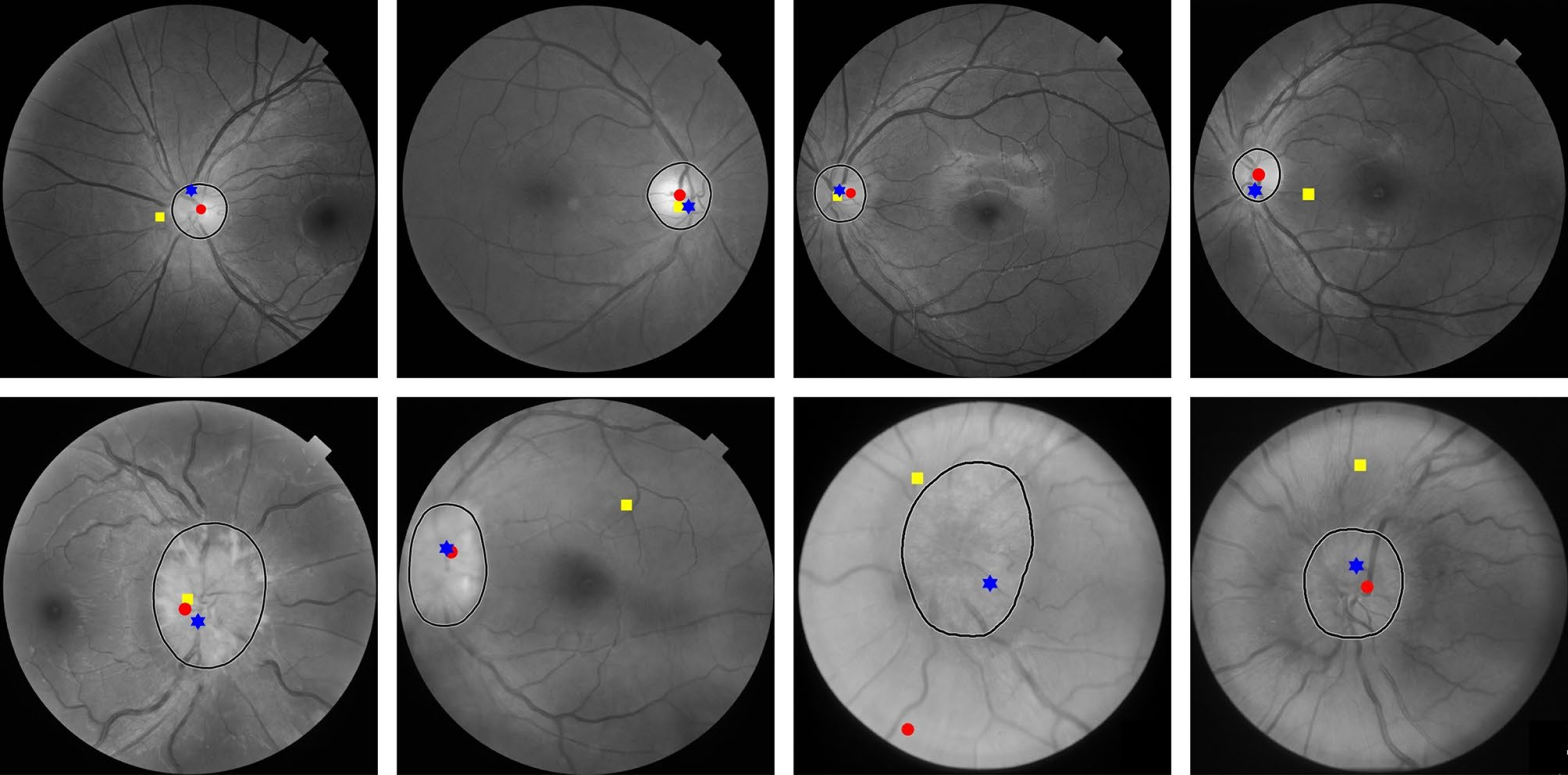
Examples of OD localization results of edematous cases from FP (yellow square), AT (red circle), and HLM used by the FGVF method (blue hexagram) for non-edematous (top) and edematous (bottom).

We compared the hybrid localization method (HLM)^24^ used by our method against the feature projection (FP)^22^ and adaptive thresholding (AT)^10^ methods. Selected cases of localization results from non-edematous and edematous groups of two datasets are depicted in Fig. 6.

The numerical results are reported in Table 3. For non-edematous ODs, most methods could locate the OD efficiently. The FP performed worse than other methods because it relied only on vessels. When the vascular network was incomplete in some edematous cases, the FP failed. AT could sometimes spot abnormally high bright spots as the OD.

**Table 3:** Comparison of the OD localization performance of FP, AT, and HLM.

Methods	Datasets	*Acc*_*loc*_		*Average Acc*_*loc*_ (%)
		Non-edematous	Edematous	
FP^22^	Internet	97.37	74.29	85.83
	RFMiD	92.31	93.41	92.86
	RFMiD2.0	100.00	55.00	77.50
	Average	96.56	73.23	85.40
AT^10^	Internet	97.37	77.14	87.67
	RFMiD	100.00	98.90	99.45
	RFMiD2.0	100.00	75.00	87.50
	Average	99.12	83.68	91.54
HLM^24^ (ours)	Internet	100.00	97.14	98.63
	RFMiD	100.00	100.00	100.00
	RFMiD2.0	100.00	90.00	95.00
	Average	**100.00**	**95.71**	**97.88**

The highest number in class is bold.

Results from Fig. 6 and Table 3 showed that the HLM method used by our algorithm achieved the best average *Acc*_*loc*_ of 97.88% for all three datasets and was considerably higher than the FP and AT methods. The *Acc*_*loc*_ values of all three methods were lower in the edematous cases than in the non-edematous cases. Across all OD types, the average *Acc*_*loc*_ of HLM was higher than FP and AT by 12.48% and 6.04%, respectively. Moreover, HLM localization performance was significantly superior to the other two comparative methods, especially in edematous cases. For such cases, *Acc*_*loc*_ of HLM was higher than FP and AT by as much as 22.49% and 12.03%, respectively.

### OD segmentation

**Figure 7:**
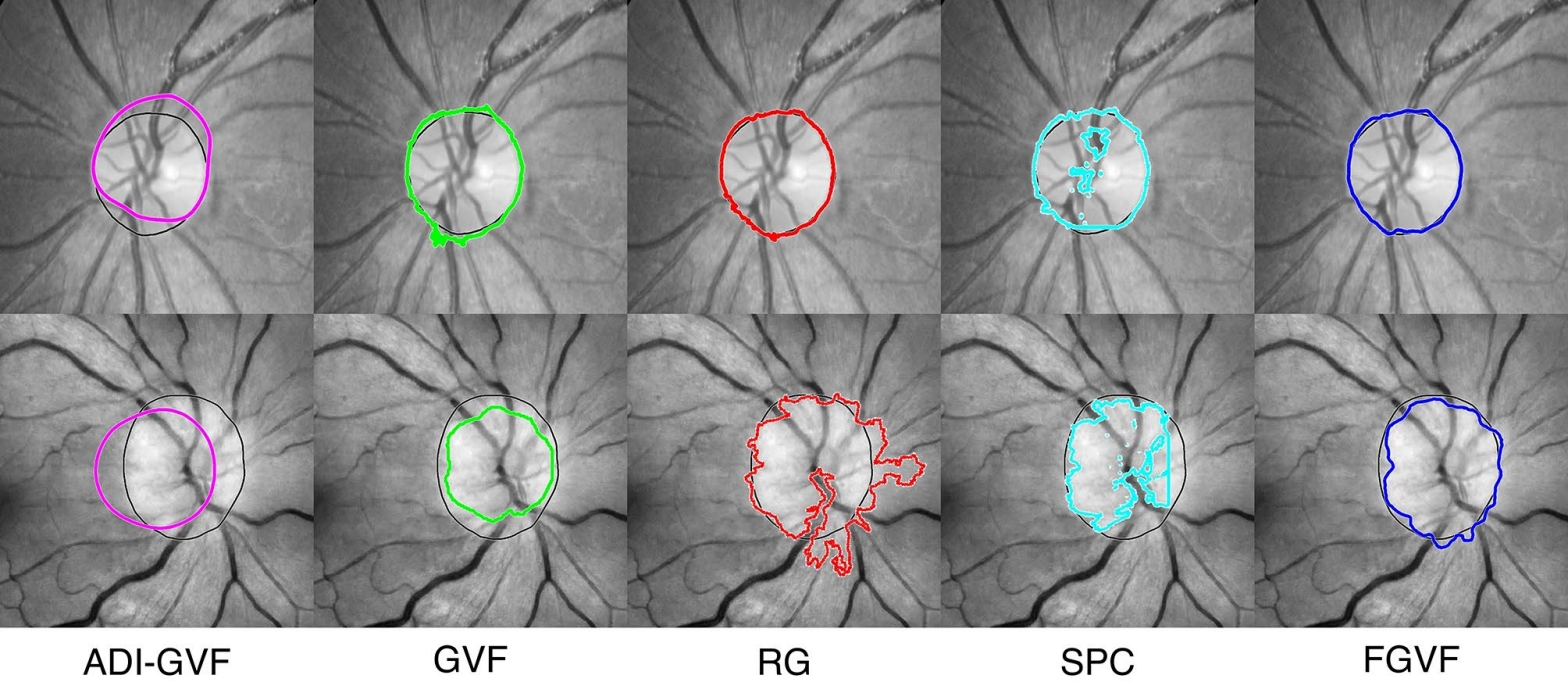
Examples of segmentation results of non-edematous cases (top) and edematous (bottom) for ADI-GVF, GVF, RG, SPC, and FGVF (ours).

We compared the factorized gradient vector flow (FGVF)^8,9^ used in our work against four other comparative methods: alternated deflation-inflation gradient vector flow (ADI-GVF)^37^, traditional gradient vector flow (GVF)^36^, region growing (RG)^29^, and super-pixel clustering (SPC)^30^. All methods except super-pixel clustering required initial points. The OD locations obtained from the HLM method were the initial points. Figure 7 shows examples of segmentation results from different approaches for edematous and non-edematous ODs.

Most methods performed better on the non-edematous ODs than the edematous ODs. For edematous OD, the methods in the GVF family showed undersegmentation, while the region growing and superpixel clustering showed oversegmentation. Most methods worked well for non-edematous OD. Table 4 shows the numerical performance comparison of segmentation methods.

**Table 4:** OD segmentation performance.

Type	Dataset	Method	Performance (%)		
			Precision	Recall	F1 measure
Non-edematous	Internet	ADI-GVF	64.77	74.83	69.44
		GVF	89.01	**96.47**	**92.59**
		RG	89.77	88.10	88.93
		SPC	89.38	76.69	82.55
		FGVF (ours)	**96.55**	86.06	91.00
	RFMiD	ADI-GVF	51.41	**99.44**	67.78
		GVF	90.51	96.48	93.40
		RG	93.36	84.17	88.53
		SPC	78.73	79.25	78.98
		FGVF (ours)	**92.97**	94.67	**93.81**
	RFMiD2.0	ADI-GVF	64.40	**99.74**	78.27
		GVF	**99.07**	89.53	94.06
		RG	87.70	82.73	85.14
		SPC	98.77	72.06	83.33
		FGVF (ours)	98.79	93.66	**96.16**
	**Average**	ADI-GVF	60.19	91.34	72.56
		GVF	92.86	**94.16**	93.51
		RG	90.28	85.00	87.56
		SPC	88.96	76.00	81.97
		FGVF (ours)	**96.10**	91.46	**93.72**
Edematous	Internet	ADI-GVF	75.31	74.04	74.67
		GVF	90.62	76.90	83.20
		RG	50.58	**85.42**	63.54
		SPC	83.08	68.70	75.21
		FGVF (ours)	**91.74**	79.21	**85.02**
	RFMiD	ADI-GVF	74.68	77.04	75.84
		GVF	**95.01**	70.72	81.08
		RG	66.13	**86.60**	74.99
		SPC	84.24	77.48	80.71
		FGVF (ours)	84.64	84.47	**84.55**
	RFMiD2.0	ADI-GVF	56.94	76.57	65.31
		GVF	66.22	69.77	67.95
		RG	43.12	80.23	56.09
		SPC	**95.20**	42.57	58.83
		FGVF (ours)	54.66	**91.05**	**68.31**
	**Average**	ADI-GVF	68.98	75.88	72.27
		GVF	83.95	72.46	77.78
		RG	53.28	84.08	65.23
		SPC	**87.51**	62.92	73.21
		FGVF (ours)	77.01	84.91	**80.77**
All type combined	Internet	ADI-GVF	70.04	74.44	85.56
		GVF	89.82	86.69	87.90
		RG	70.18	**86.76**	76.24
		SPC	86.23	72.70	78.88
		FGVF (ours)	**94.15**	82.64	**88.01**
	RFMiD	ADI-GVF	63.05	88.24	71.81
		GVF	**92.76**	83.60	87.24
		RG	79.75	85.39	81.76
		SPC	81.49	78.37	79.85
		FGVF (ours)	88.81	**89.57**	**89.18**
	RFMiD2.0	ADI-GVF	60.67	88.16	71.79
		GVF	82.65	79.65	81.01
		RG	65.41	81.48	70.62
		SPC	**96.99**	57.32	71.08
		FGVF (ours)	76.73	**92.36**	**82.24**
	**Average**	ADI-GVF	64.59	83.61	72.88
		GVF	**88.41**	83.31	85.78
		RG	71.78	84.54	77.64
		SPC	88.24	69.46	77.73
		FGVF (ours)	86.56	**88.19**	**87.37**

*The highest is bold.

The following findings can be summarized from the results of Table 4.

In the case of non-edematous images, both GVF and FGVF methods have F1 measures that are significantly higher than other comparative methods. On average, the improvement of FGVF over the second-best method (GVF) is only 0.21%. However, FGVF outperforms the poorest method (ADI-GVF) by 21.16%.

It was found that for images with edema, all methods performed worse than those without edema. Among all the methods, FGVF was the best and had significantly better results than GVF and other methods. On average, the improvement of FGVF over the second-best method (GVF) was 2.99%, while the improvement of FGVF over the poorest method (RG) was 15.54%.

In general, regarding mix cases, both GVF and FGVF have F1 measure values that are fairly close, but significantly better than other methods. Precision-wise, GVF was slightly better than FGVF, but FGVF had considerably better recall than GVF. This resulted in FGVF having a better overall F1 measure than GVF. However, the ADI-GVF method was the poorest performer among them.

The RFMiD2.0 dataset is known to be more challenging for most methods due to the low resolution and indistinct OD region in edematous OD images. However, when only considering edematous OD images in this dataset, FGVF still performs best.

### Edema classification

Table 5 compares the linear SVM classifier accuracy of classification performance using each sole feature set and combined feature sets on different datasets.

**Table 5:** Accuracy comparisons of the proposed method (all featured combined) against each feature set and also against a state-of-the-art method (Yousaf et al.^7^).

Methods	Feature type	*Acc*_*classify*_ (%)			*Average Acc*_*classify*_ (%)
		Internet	RFMiD	RFMiD2.0	
	GLCM	84.90	84.10	95.00	88.00
	Vessel	89.00	94.50	97.50	93.67
	Color	90.40	87.40	100.00	92.60
	Intensity line profile	**100.00**	97.80	92.50	96.80
Proposed	All types combined	98.60	**99.50**	**100.00**	**99.40**
Yousaf et al.^7^		95.90	94.50	97.50	95.97

*The highest number in class is bold.

The findings and discussions from Table 5 are as follows.

**Figure 8:**
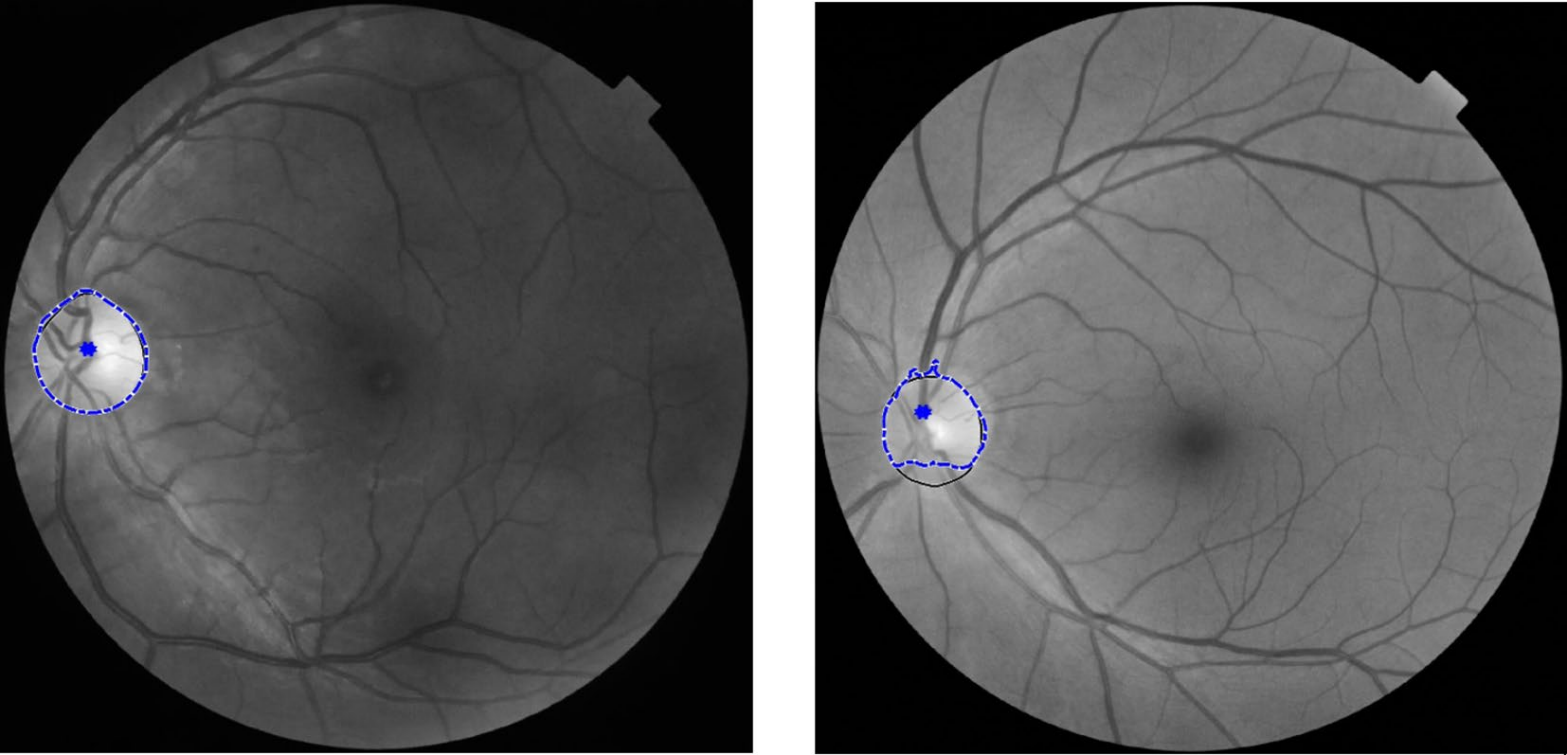
Examples of false negative (left) and false positive (right) cases. The black solid contour is the ground truth, the hexagon is the OD location, and the dash line is the OD boundary.

1. The proposed method achieved an average accuracy of 99.40%, which was the highest accuracy recorded.
2. The results of the average accuracy classification for a single feature type showed that the intensity line profile, vessel, color, and GLCM gave the best to worst results, respectively. However, in the proposed work, the average accuracy significantly improved. The overall improvement of all feature types combined compared to the best type (the intensity line profile) was 2.6%, while compared to the worst type (GLCM) was 11.4%.
3. It should be noted that our proposed method had shown better performance than the method proposed by Yousaf et al.^7^ by 3.34%. This improvement could be attributed to the fact that Yousaf et al. used only ten features from vessels and GLCM, while our method also employs features from the Color and intensity line profiles. This suggests that using additional features could help improve the classification results.
4. After analyzing the unsuccessful cases, we found that the accuracy of classification depended on several factors, such as the stages of edema in the dataset and the appearance of the OD. We noticed that when the images dealt with the mild edema stage in the dataset, the classification accuracy was lower. This was because the differences in characteristics between mild edema and normal OD showed minimal changes in the appearance of the disk. Additionally, some non-edematous OD with unclear boundary resulted in incorrect segmentation of the OD, which led to extracting wrong features and consequently resulted in inaccurate classification. Figure 8 shows examples of an edematous OD misclassified as non-edematous (false negative) and a non-edematous image misclassified as edematous (false positive).

## Conclusion

This paper presents an automatic classification and segmentation of optic disks with edematous and non-edematous based on the FGVF segmentation model using HLM initialization and classification results from a linear SVM classifier. The proposed method was evaluated on 146 edematous and 149 non-edematous images from Internet and RFMiD datasets by comparing the proposed localization, segmentation, and classification performances against the existing methods. The HLM worked well for OD localization and correctly located the OD in 295 out of 292 images with 97.88% accuracy. The proposed FGVF achieved an average segmentation precision of 86.56%, recall of 88.19%, and F1-score of 86.48%. The average classification accuracy was 99.40%. However, the FGVF method used in the OD segmentation algorithm had limitations, including high computational demands and sensitivity to initial conditions. For edematous OD classification, accuracy relied heavily on the precision of OD segmentation. Finding more useful features, such as the cloud OD boundary and the ratio of OD diameter to that of the retina, and improving the limitations of FGVF will be our future work.

## Data Availability

The datasets used in the current study are available in Google Drive through the provided link. https://drive.google.com/drive/folders/1vgHmgvxkFtU4m7NZ4IbZWwCzenLh7xXG.
